# The Psychometric Properties of the Cone Evasion Walk Test in Patients With Knee Osteoarthritis

**DOI:** 10.1155/oti/3339937

**Published:** 2026-07-13

**Authors:** Kevser Sevik Kacmaz, Bayram Unver, Vasfi Karatosun

**Affiliations:** ^1^ Department of Physiotherapy, Kayseri University, Incesu, Kayseri, Türkiye; ^2^ Faculty of Physical Therapy and Rehabilitation, Dokuz Eylul University, Balçova, Izmir, Türkiye, deu.edu.tr; ^3^ Department of Orthopaedics and Traumatology, School of Medicine, Dokuz Eylul University, Balçova, Izmir, Türkiye, deu.edu.tr

**Keywords:** balance, falls, osteoarthritis, performance, reliability, validity

## Abstract

**Background:**

Knee osteoarthritis can impair mobility, gait, and balance and increase the risk of falls. The Cone Evasion Walk Test (CEW) is a comprehensive test that evaluates mobility, dynamic balance, and fall risk by assessing the ability to walk and turn in all directions while evading obstacles. It incorporates cognitive, sensory, visual, and various neuromusculoskeletal functions. This study was aimed at estimating the validity, reliability, and feasibility of the CEW in patients with knee osteoarthritis.

**Methods:**

Thirty‐six patients with knee osteoarthritis underwent two CEW trials on the same day. The Timed Up and Go Test and the Western Ontario and McMaster Universities Arthritis Index (WOMAC) were used. The intraclass correlation coefficient (ICC 2,1), standard error of measurement (SEM95), and smallest detectable change (SDC95) were calculated to assess reliability and measurement precision. Spearman′s correlations determined concurrent validity. The feasibility of the CEW was assessed based on completion rate, adherence, acceptability, practicality, and adverse effects.

**Results:**

The CEW demonstrated almost perfect within‐day (intrasession) reliability and moderate concurrent validity (*r* = 0.58 [95% CI: 0.317, 0.766], *p* < 0.001). The ICC (2,1), SEM95, and SDC95 for the CEW were 0.97 (0.87–0.99), 0.73 (0.59–0.95), and 2.02 (1.63–2.63), respectively, with 95% CI. The CEW was also found to be highly feasible.

**Conclusion and Implications of Key Findings:**

The findings support the use of the CEW to assess patients with knee OA. Our analysis revealed within‐day (intrasession) reliability, moderate concurrent validity, and high feasibility of the CEW. Given its convenience, short administration time, and minimal space and equipment requirements, the CEW may be used by occupational therapists as a complementary performance‐based measure within a broader occupation‐centered assessment to identify mobility‐related safety barriers and monitor interventions aimed at supporting participation in daily activities and community mobility.

## 1. Introduction

Knee osteoarthritis (OA) is a prominent cause of disability in individuals, causing significant functional impairment and restrictions in daily activities [1]. It leads to various dysfunctions, such as impaired proprioception, altered neuromuscular activation, reduced knee strength and range of motion, and impaired ability to walk and avoid obstacles; proactive gait control; reaction time; postural instability; and balance, which predispose them to an increased risk of falling [2]. Almost half of all deaths related to injuries can be attributed to falls among the elderly [3]. Impaired postural stability and balance are among the primary causes of falls, disrupting daily living and mobility [4].

One of the leading causes of falls is tripping while navigating obstacles. Almost 53% of falls occur due to tripping while walking [5]. Successfully clearing obstacles demands a delicate balance between the precise movement of the trailing foot and maintaining body balance on the supporting leg [6]. Patients with knee OA may have an elevated risk of falls when navigating obstacles due to the stringent demands placed on the lower extremities. As a result, individuals with knee OA face a heightened risk of falling, which is substantially increased if they experience joint pain and have a history of falls [7]. Additionally, factors such as the presence of valgus deformity and joint laxity further deteriorate proprioception and increase balance deficits and fall risk in patients with knee OA. Thus, evaluating walking, balance, and fall risk with valid and reliable objective assessment tools is necessary in patients with knee OA [8].

Currently, there is no universally agreed‐upon screening test to identify walking and balance problems associated with fall risk [9]. A few tests assess walking with attentional and proactive gait control, such as the TUG‐dual (the Timed Up and Go dual‐task test), which demands negotiating, turning, and walking. However, it does not include turning in all directions or navigating obstacles [10]. The Cone Evasion Walk Test (CEW) was developed to evaluate *proactive gait adaptability during obstacle negotiation* (i.e., the ability to adjust the walking trajectory and stepping pattern to evade obstacles that require multidirectional turns). This test incorporates cognitive, sensory, visual, and various neuromuscular and motor functions [11]. From the standpoint of occupational therapy, engagement in everyday and instrumental activities of daily living is directly linked to safe and adaptable mobility [12]. Moving through home and community contexts, changing direction, negotiating furniture and other obstacles, and adjusting their gait to environmental demands are all common tasks in everyday activities [13]. For those with knee OA who may have trouble with movement, self‐care, and everyday activities, these criteria could be more difficult. The CEW may provide occupational therapists with performance‐based information on mobility‐related obstacles to occupational performance, as it evaluates multidirectional turning and obstacle avoidance under standardized conditions. This data may be used to supplement self‐report assessments and to aid in planning interventions involving the use of assistive devices, task and environmental adjustments, fall prevention techniques, and functional change monitoring.

Objective evaluations empower the rehabilitation team to establish and tailor goals, as well as modify and appraise their efforts effectively [14]. Although these performance tests are validated for evaluating gait and balance in various groups, the calculated psychometric properties are specific to that population [8]. Therefore, determining the CEW′s reliability and validity in patients with knee OA is necessary. To date, no studies have examined the reliability and validity of the CEW in patients with knee OA. Thus, the present study was aimed at assessing the validity, reliability, and feasibility of the CEW in patients with knee OA.

We hypothesized that the CEW would be a feasible, reliable, and valid performance‐based test for assessing functional mobility and dynamic balance in patients with knee OA. Specifically, we expected that:

- Patients with knee OA would be able to complete the CEW safely and without adverse effects, supporting its feasibility,
- The CEW would demonstrate excellent test–retest reliability, indicating consistent performance across repeated trials, and
- The CEW completion time would show a significant positive correlation with the Timed Up and Go Test (TUG), supporting its concurrent validity as a measure of functional mobility and dynamic balance.

## 2. Methods

### 2.1. Design

This study utilized a cross‐sectional design. An ethics committee approved the study before its commencement, with Approval Number 2020/03‐21 and date 03.02.2020. Before participating in the study, each patient provided written informed consent, as required by ethical standards, including the Declaration of Helsinki and its subsequent revisions.

### 2.2. Patients

The determination of the required minimum sample size was performed using G∗Power software [15]. Power analysis indicated that at least 28 subjects were needed to achieve a minimum reliability coefficient of 0.75 with *α* = 0.05 and power = 0.80.

Thirty‐six patients were included. Participants were eligible for inclusion if they were 18 years of age or older, had degenerative knee OA, and could maintain independent mobility. Although knee OA is more commonly observed in individuals aged 50 years and older, the age criterion was kept broad to include all adult patients with a confirmed clinical diagnosis. The exclusion criteria were inability to understand the study instructions or another pain, disorder, or surgery in the lower extremities that may affect gait or balance.

Patients diagnosed with knee OA by an orthopedic and traumatology physician and who attended routine follow‐ups were invited to participate in the study at the orthopedic and traumatology clinic where the research was conducted. All eligible and voluntary patients were included in the study consecutively without researcher selection, between May 2021 and December 2023, on occasions when the assessor was available at the clinic where this study took place.

### 2.3. Protocols

Demographic and clinical data, including history of falls over the last 12 months, pain levels, and scores on the Western Ontario and McMaster Universities Arthritis Index (WOMAC), CEW, and TUG, were obtained.

The same physiotherapist, with years of professional experience in assessing and rehabilitating orthopedic patients, demonstrated the CEW and TUG to the patients. No additional education was required, as the tests were simple and easy to understand. To reduce variability in both environmental conditions and measurement procedures, identical instructions were administered to all participants. Additionally, to standardize performance, reduce initial unfamiliarity, and minimize potential learning effects, participants completed two practice trials before timed assessments.

The setting was an outpatient orthopedics and traumatology clinic of a regional university hospital. The floor was covered with straight tiles, and the environment was well lit. All evaluations were administered under identical physical conditions in the hospital′s clinical room.

Patients underwent CEW trials twice on the same day, with a 1‐h interval between trials. Patients sat during this period to prevent fatigue. Previous studies examining the reliability of performance‐based measurement methods have reported that a 1‐h resting period between trials is sufficient to reduce the influence of fatigue and minimize learning effects [16, 17]. A 5‐min rest or more was permitted between the tests if needed.

To assess pain, the Visual Analog Scale (VAS) was administered immediately before and after the performance‐based assessments. The patients also completed the WOMAC and performed the TUG to examine concurrent validity.

### 2.4. Outcome Measures

The following outcome measures were used to evaluate patients.

#### 2.4.1. The CEW

**Figure 1:**
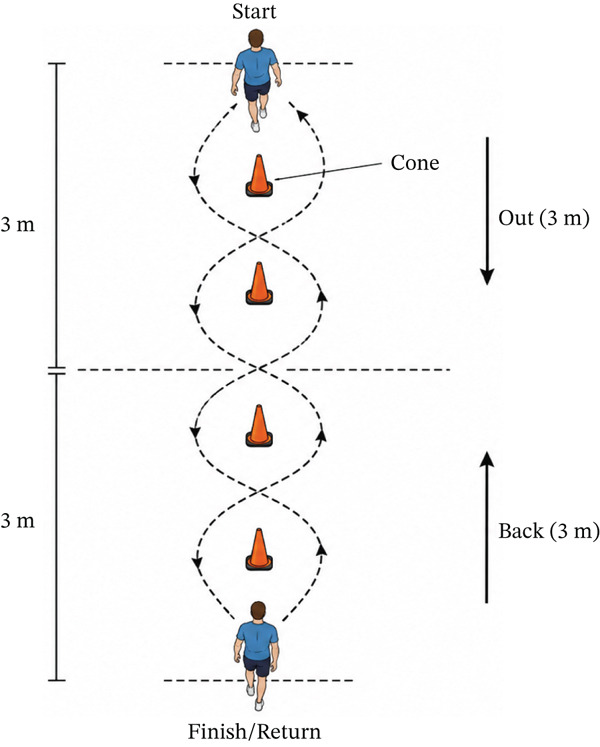
The Cone Evasion Walk Test.

The CEW is an innovative functional performance test that involves traversing 3 m twice and navigating around four cones without touching them. These cones are strategically positioned along a centerline. Depending on whether the individual walks unaided (5 cm), with crutches or a cane (5 cm), a walker (25 cm), or a walking table (30 cm), the distance between the cones and the center line varies. Participants perform the test at their chosen pace, using their regular walking aids and, if necessary, receiving support from a walking aid, as documented in the test assessment notes. The examiner records the time taken to finish the test and the count of cones touched on either side [11]. An illustration of the CEW was shown in Figure 1.

#### 2.4.2. TUG

Participants were instructed to rise from a chair, walk as rapidly as possible to a line marked 3 m away, return to the chair, and sit back down. The subjects′ initial positions were standardized, with attention to buttock placement, back support, hand use, and foot positioning. The seat height for the test was set at a standard 47 cm (18.5 in.). All assessments were conducted using the same chair to maintain uniform testing conditions [18].

#### 2.4.3. The WOMAC

The WOMAC is a widely used questionnaire that measures stiffness, function, and pain related to OA of the lower extremities by assessing 17 functional activities, five pain‐related activities, and two categories of stiffness. The tool has demonstrated high levels of reliability and validity in evaluating patients with knee OA [19].

#### 2.4.4. VAS

The VAS has a 10‐cm horizontal line. Patients are asked to mark a line corresponding to their pain levels. A score of 0 represents the absence of pain, whereas a score of 10 denotes the most severe pain imaginable [20].

### 2.5. Statistical Analysis

The Kolmogorov–Smirnov test, skewness, kurtosis, standard deviation, and a histogram were used to assess the normality of the data distribution. The data were analyzed using parametric or nonparametric methods depending on their distribution.

A paired‐sample *t*‐test was used to investigate differences in pain levels before and after the tests. The absolute agreement model of the intraclass correlation coefficient (ICC 2,1), two‐way random single measures (absolute agreement) at the 95% confidence level, was used to determine within‐day (intrasession) reliability [17]. The ICC (2,1) was interpreted according to the following cutoff points suggested by Bland and Altman: *poor* (< 0.20), *fair* (0.21–0.40), *moderate* (0.41–0.60), *good* (0.61–0.80), and *excellent* (≥ 0.81) [8]. The standard error of measurement (SEM95) was computed to assess the precision of the assessment method at the 95% confidence level [8].

The smallest detectable change at the 95% confidence level (SDC95) was acquired by multiplying the point for the SEM by the *z* = 1.95 for the 95% confidence interval by √2 [17].

Spearman′s correlations were calculated to determine the concurrent validity. A correlation coefficient between 0 and 0.30 is considered *poor*, 0.31–0.50 is considered *fair*, 0.51–0.70 is considered *moderate*, 0.71–0.90 is considered *good*, and > 0.90 is considered *excellent* [8].

All data were analyzed using IBM SPSS Statistics (Version 20.0). A *p* value of < 0.05 was considered statistically significant.

## 3. Results

Thirty‐six patients (28 females and eight males) with knee OA participated in this study. The mean age was 61.76 ± 10.48 years, and the mean BMI was 30.38 ± 4.21.

Thirty‐one of the patients have bilateral knee OA, and five have unilateral knee OA. All five patients with unilateral knee OA have right‐leg dominance. Four of them had knee OA on the right leg, and one of them had knee OA on the left leg.

According to the Kellgren–Lawrence classification, 14 (38.8%) patients had Level 2, 19 (52.7%) had Level 3, and 4 (8.3%) had Level 4.

During the performance tests, two patients used canes, and one patient used a walker.

The demographic and clinical features of the patients are demonstrated in Table 1. The mean completion time of the CEW was 10.75 ± 3.30 s. The mean WOMAC score was 49.30 ± 25.32, and the TUG score was 11.68 ± 6.01. The average number of falls in the last 12 months was 0.42 ± 0.67.

**Table 1:** The demographic characteristics of the patients.

Variables	*M* *e* *a* *n* ± *s* *t* *a* *n* *d* *a* *r* *d* *d* *e* *v* *i* *a* *t* *i* *o* *n*	Min–max
Age (years)	61.76 ± 10.48	30–84
Body mass index (kg/m^2^)	30.38 ± 4.21	18.21–39.68
Weight (kg)	81.60 ± 14.80	47–112
Height (cm)	163.91 ± 7.27	153–187

Abbreviations: kg, kilogram; m^2^, square meter.

In this study, the CEW demonstrated excellent within‐day (intrasession) reliability and moderate concurrent validity (*p* < 0.001). The relative (ICC 2,1) and absolute (SEM95 and SDC95) reliability of CEW are 0.97 (0.87–0.99), 0.73 (0.59–0.95), and 2.02 (1.63–2.63), respectively, with 95% CI, as shown in Table 2. The correlation between the CEW and TUG is 0.58 (95% CI: 0.317, 0.766, *p* < 0.001; Table 3).

**Table 2:** The relative and absolute reliability of the Cone Evasion Walk Test.

Variables	First trial (*m* *e* *a* *n* ± *S* *D*)	Second trial (*m* *e* *a* *n* ± *S* *D*)	Difference (*m* *e* *a* *n* ± *S* *D*)	ICC 2,1 (95% CI)	SEM (95% CI)	SDC (95% CI)
CEW (s)	10.75 ± 3.30	10.56 ± 3.70	0.18 ± 0.20	0.97 (0.87–0.99)	0.73 (0.59–0.95)	2.02 (1.63–2.63)

Abbreviations: CEW, Cone Evasion Walk Test; CI, confidence interval; ICC, intraclass correlation coefficient; SEM, standard error of measurement; SD, standard deviation; SDC95, smallest detectable change.

**Table 3:** The correlations among functional performance measures and the Cone Evasion Walk Test.

Variables	CEW	
	*r*	*p*
TUG score 95% CI	**0.585** ^∗^ (0.317–0.766)	**0.001** ^∗^
WOMAC score 95% CI	0.297 (−0.035 to 0.570)	0.117
History of falls (number) 95% CI	0.112 (−0.225 to 0.425)	0.556

*Note:* Bold text indicate statistical significance.

Abbreviations: CEW, Cone Evasion Walk Test; TUG, Timed Up and Go Test; WOMAC, Western Ontario and McMaster Universities Arthritis Index.

^∗^ *p* < 0.05, Spearman′s correlations.

The second CEW trial was completed 0.18 ± 0.20 s faster by the patients than the first; however, this difference was not statistically significant (*p* > 0.05). Only one patient touched a cone one time. Nearly all participants completed the procedure without difficulty, and no significant problems arose. All test trials were retained for analysis; none were disqualified. Mean VAS scores showed no significant change between pretest and post‐test assessments, and no adverse effects related to the performance tests were observed. The tests were well tolerated and completed efficiently by the patients. Therefore, the CEW was found to be feasible in patients with knee OA.

## 4. Discussion

This study was aimed at assessing the validity, reliability, and feasibility of the CEW in patients with knee OA. The CEW showed excellent within‐day (intrasession) reliability and moderate concurrent validity in this study. This study also provided values for SEM95 and SDC95 for the CEW, which can be helpful in clinical trials and practice. The SDC95 of 2.02 s indicates that performance alterations exceeding this value are likely real. Patients′ pain levels did not increase at the end of the trials, and no adverse events related to the DFT were observed, suggesting that the CEW was well tolerated.

The CEW was developed to assess proactive gait adaptability during obstacle negotiation in response to environmental challenges that frequently occur in daily life [11]. Other performance‐based fall risk and walking tests, such as the TUG, do not include turning in all directions or evading physical obstacles. The CEW is the only available performance test that requires turning and directional changes in all directions while evading obstacles. Therefore, it is a more comprehensive test that can detect a broader range of aspects related to patients′ fall risk, walking, dynamic balance, proactive gait control, and cognitive abilities.

The CEW was found to have excellent within‐day (intrasession) reliability, which estimates the consistency across time intervals in the absence of any change in the individual′s health status [21]. This reliability may be due to the test′s objective nature, well‐designed structure, and clear instructions. Additionally, a 1‐h rest period was provided to prevent fatigue‐related effects, and two practice trials were conducted to diminish learning effects. However, to date, only a few studies have investigated the CEW′s reliability and validity. Sevik Kacmaz et al. (2023) found similar results. They reported excellent reliability in patients with hip OA (ICC 2, 1 = 0.96) [22]. Sjöholm et al. reported high–excellent reliability in patients with stroke (ICC 2, 1 = 0.88 − 0.98) [11]. Therefore, these studies support the current study′s findings and confirm that the CEW is a reliable tool for proactive gait adaptability during obstacle negotiation in patients with knee OA. This may be due to the CEW being a well structured and detailed test with clear instructions.

In this study, SEM95 was used to quantify measurement error, and SDC95 was used to determine the minimum clinically significant difference. A score of 2.02 or above on the CEW is considered a fundamental change in a patient′s condition [21]. Values of 0.73 (SEM95) and 2.02 (SDC95) indicate that the CEW is very sensitive and has a low risk of error. These results suggest that the CEW may easily catch even minor alterations in the clinical status of patients with knee OA. Therefore, the CEW appears to be a valuable, performance‐based method for sensitive, accurate assessments in clinical practice and research. Additionally, this feature may be especially significant for those in the early stages or with mildly affected knees, where the pathology′s impact is minimal, and other tests cannot detect the subtle differences.

The concept of feasibility refers to both the acceptability of the assessment method to respondents and its operational viability within the evaluation. When assessing health state preferences, feasibility is commonly determined by analyzing completion rates [23]. In this study, all patients completed the CEW without any significant issues. The CEW did not increase patients′ pain levels, and no adverse effects were observed during the performance tests. Therefore, the CEW was found to be a feasible test.

The CEW had a moderate relationship with the TUG. While they correlate, the physical abilities required for the TUG and CEW tests are not identical. The TUG primarily measures gait speed, a single aspect of mobility that does not assess the balance required to perform various tasks in independent activities of daily living. Therefore, the moderate correlation with TUG reflects partial overlap in functional mobility/gait speed, while the CEW′s additional demands for obstacle avoidance and turning represent *complementary domains* not captured by the TUG. Hence, the CEW can be viewed as a more comprehensive assessment of balance and mobility, essential for activities of daily living. However, the CEW and WOMAC did not correlate. The lack of correlation with WOMAC is as expected, given the divergence between performance‐based and self‐reported outcomes, supporting complementary rather than redundant measurement domains. This further emphasizes the significance of performance‐based tests in patient evaluation, where “what the patient can do” and “what the patient thinks he/she can do” may differ. To accurately identify deficits in functional mobility and balance, a more thorough and multidimensional evaluation can be achieved by employing both methods [11, 24]. There are only two articles in the literature regarding the validity of the CEW. Sjöholm et al. reported significant correlations between the CEW and few functional performance tests, such as functional ambulation classification (*r* = −0.67), TUG (*r* = 0.45), Montreal Cognitive Assessment (*r* = −0.36), Star Cancellation Test (*r* = −0.36), and number of falls in the next 6 months (*r* = 0.18, *p* = 0.01) [11]. Sevik Kacmaz et al. (2023) also studied the validity of the CEW in patients with hip OA. They also found a moderate correlation between the CEW and the TUG (*r* = 0.74) [22], which is similar to both our population and the findings.

The occupational therapy relevance of these findings lies in the relationship between adaptable mobility and participation in everyday occupations. Occupational therapists may investigate how mobility constraints affect activities, including moving between rooms, navigating kitchens and bathrooms, shopping, and accessing community areas, in response to a prolonged CEW completion time or difficulties in avoiding barriers. Therefore, occupation‐focused intervention design, such as task‐specific mobility practice, environmental modification, assistive device training, activity pacing, and fall prevention techniques, may benefit from CEW findings. Occupational therapists may find it useful to track improvements in adaptable mobility across interventions using the SDC95 value found in this study.

Still, this study shows that the CEW did not correlate with the past falls in knee OA. Sevik Kacmaz et al. also could not identify a correlation between the CEW and self‐reported falls within the previous 12‐month period in patients with hip OA [22]. We anticipated these findings, as the patients had a low percentage of falls that did not reach statistical significance. Additionally, the patients′ fall histories were obtained from their self‐reports. This may be problematic due to memory issues and recall bias, which could hinder the accurate assessment of the correlation between these factors. In Sjöholm et al.′s study, Cox regression analysis indicated that individuals who contacted between four and eight cones exhibited an increased risk of future falls compared with those who touched none [11]. This study differs in that it used a different data‐collection approach and recorded future falls.

### 4.1. Limitations

This study also has some limitations. First, both CEW trials were administered on the same day with a 1‐h interval; therefore, our findings primarily reflect within‐day (intrasession) repeatability rather than day‐to‐day test–retest reliability. Same‐day testing may overestimate reliability by reducing between‐day variability and by limiting clinical fluctuations that may occur across days. Although a resting period and practice trials were used to minimize fatigue and learning effects, the second trial was completed slightly faster than the first (mean difference: 0.18 s), suggesting that a residual practice/familiarization effect may still have occurred. Future studies should confirm CEW reliability using longer retest intervals (e.g., 1–2 weeks) and evaluate stability under routine clinical follow‐up conditions. Additionally, we did not record the symptom duration of the patients, which may significantly affect their walking skills, balance, fall risk, and test results.

Moreover, the patients′ fall histories were obtained through self‐report. This may be problematic due to memory problems and recall bias. Future studies may collect fall counts by reviewing patients′ journals or conducting routine phone calls.

## 5. Conclusions

The analysis revealed that the CEW exhibits excellent within‐day (intrasession) reliability, moderate concurrent validity, and high feasibility. Therefore, the CEW can comprehensively evaluate proactive gait adaptability during obstacle negotiation in patients with knee OA. The CEW can be used as one part of a more comprehensive occupation‐centered assessment in occupational therapy practice to identify mobility‐related safety barriers that may limit participation in daily activities and community mobility. The results of the CEW can guide future assessment and intervention planning for task‐specific training, environmental modification, assistive device use, and fall prevention; however, they should be interpreted alongside self‐reported occupational performance and direct observation of activities in relevant everyday contexts.

## Funding

No funding was received for this manuscript.

## Ethics Statement

This study was approved by the Dokuz Eylul University Noninvasive Research Ethics Committee (2020/03‐21).

## Conflicts of Interest

The authors declare no conflicts of interest.

## Data Availability

The data that support the findings of this study are available upon request from the corresponding author. The data are not publicly available due to privacy or ethical restrictions.
